# Emergency Department Point-of-care Ultrasound Identification of Suspected Lemierre’s Syndrome: A Case Report

**DOI:** 10.5811/cpcem.1245

**Published:** 2023-08-09

**Authors:** Paul Craven, Bradley End, Peter Griffin

**Affiliations:** West Virginia University, Department of Emergency Medicine, Morgantown, West Virginia

**Keywords:** Case report, septic thrombophlebitis, Lemierre’s syndrome, point-of-care ultrasound

## Abstract

**Introduction:**

Lemierre’s syndrome is septic thrombophlebitis of the internal jugular vein, most commonly associated with head and neck infections. While central catheters are associated with venous thromboembolism and catheter-associated bacterial infections, cases of Lemierre’s syndrome caused by central catheters are extraordinarily rare.

**Case Report:**

We detail a case of Lemierre’s syndrome resulting from a peripherally inserted central catheter in a pregnant female patient. Diagnosis of this rare and potentially life-threatening disease process was expedited using point-of-care ultrasound.

**Conclusion:**

Diagnosis of rare but potentially life- or limb-threatening pathologies is paramount to the successful practice of emergency medicine. Identifying these rare disease processes requires a high index of suspicion and a work-up strategy that includes consideration of medical history in combination with lab and imaging findings to determine appropriate interventions.

## INTRODUCTION

Lemierre’s syndrome is septic thrombophlebitis of the internal jugular (IJ) vein. It is most commonly caused by oropharyngeal flora, usually fusobacterium species, although streptococcal species such as *Eikenella corrodens* are also common. Pathogenesis is through extension of pharyngitis, tonsillitis, odontogenic, and other oropharyngeal and head and neck infections.^1^ Septic thrombophlebitis is a known but rare complication of central catheters, with peripherally inserted central catheters (PICC) lines having reduced risk of infection.^2^ We report a case of septic thrombophlebitis related to a PICC line extending into the jugular veins and throughout the central vasculature of the chest in a pregnant patient, initially diagnosed on point-of-care ultrasound (POCUS).

## CASE REPORT

A 29-year-old female, gravida 3 para 2 at 29 weeks gestation presented to the emergency department (ED) with left arm and neck swelling. Her pregnancy had been complicated by hyperemesis gravidarum requiring a left-sided PICC. Her medical history was significant for prior pregnancies complicated by hyperemesis and a reported history of opoid use disorder on buprenorphine. The PICC had been removed at another ED approximately three days prior to presentation at our ED after the site had become erythematous and painful. She was placed on oral antibiotics and recommended to follow up with her obstetrician. Despite removal of the the PICC, the site had become severely swollen and erythematous extending over her left neck. In addition, she had begun to experience chest pain, worsening shortness of breath, fever, chills, and left arm paresthesias, which prompted her to seek evaluation.

**Image 1:**
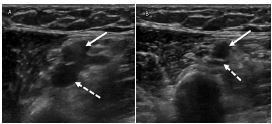
Point-of-care ultrasound of the left axilla in short axis using a 15-6 MHz linear probe: A) without compression; and B) with compression. The axillary vein (solid white arrow) demonstrates non-compressible venous structures with near-complete collapse of the concomitant artery (dotted white arrow).

**Image 2:**
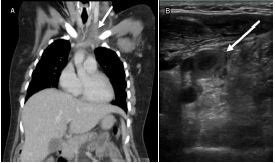
A) Contrast-enhanced computed tomography of the chest in coronal view demonstrating lack of contrast in the left internal jugular vein (arrow). B) Point-of-care ultrasound of the left neck using a 15-6 MHz linear probe demonstrating lack of compressibility of the left internal jugular vein with noted thrombus within the lumen (arrow).

On arrival her heart rate (HR) was in the 160s beats per minute (bpm) and blood pressure (BP) 95/67 millimeters of mercury (mm Hg); otherwise, her vital signs were within normal limits. On physical exam, the PICC site was erythematous, swollen, and tender. She had marked swelling involving her left upper extremity, chest, and left side of the neck. Given the patient’s vitals and physical exam, there was significant clinical suspicion for deep venous thrombosis (DVT). We used point-of-care-ultrasound to perform a bedside DVT assessment with noted extensive clot burden extending through the basilic vein into the axillary vein (Image 1), as well as in the IJ vein (Image 2). Serum labs were notable for a white blood count of 20.7 × 10^3^ cells per microliter (uL) with 80% neutrophils (reference range: 3.7–11 ×10^3^ cells/uL).

The findings prompted a recommendation that she undergo a computed tomography (CT) pulmonary embolism protocol, which she consented to. Cross-sectional imaging demonstrated extensive clot burden encompassing the left brachiocephalic, left subclavian, and left internal and external jugular veins (Image 2). The CT was also concerning for septic pulmonary emboli. Given the combination of extensive clot burden encompassing the IJ, in addition to septic emboli, we were able to confirm the diagnosis of Lemierre’s syndrome. The patient was immediately initiated on heparin and broad-spectrum antibiotics.

Obstetrics was consulted at the time of the patient’s arrival and was bedside shortly thereafter. She received dexamethasone six milligrams intramuscular in the event of an emergent cesarean section. Fortunately, neonatal stress testing demonstrated no evidence of fetal distress, and the patient continued to improve. She was subsequently admitted to the intensive care unit (ICU) by which time her HR had improved to 116 bpm and BP to 105/52 mm Hg. Interventional radiology was also consulted for possible thrombectomy vs thrombolysis at time of arrival; however, given the patient’s improvement by time of admission to the ICU the recommendation was for conservative therapy. While inpatient, blood cultures resulted positive for methicillin-sensitive *Staphylococcus aureus* (MSSA).

**CPC-EM Capsule**

What do we already know about this clinical entity?

*Lemierre’s syndrome is a bacterial infection that extends to the lateral pharyngeal space, precipitating septic thrombophlebitis of the internal jugular veins.*

What makes this presentation of disease reportable?

*Lemierre’s syndrome is rare; any opportunity to identify and learn from the disease process is beneficial, particularly expediting diagnosis.*

What is the major learning point?

*Ultrasound can be an effective modality to aid in the diagnosis of Lemierre’s syndrome.*

How might this improve emergency medicine practice?

*Ultrasound is often more readily available in the evaluation of unstable patients and can be performed in resource-limited settings.*

Ultimately the patient did very well without requiring procedural intervention or cesarean section. She was continued on enoxaparin and discharged on six weeks of cefazolin.

## DISCUSSION

Lemierre’s syndrome is a rare form of septic thrombophlebitis, usually arising from an infection of the head or neck. Our literature review revealed only two case reports of Lemierre’s syndrome resulting from catheter use. The first described a dialysis catheter in the patient’s IJ, and the second a PICC line that had migrated into the jugular vein.^3^,^4^ Our case represents a unique example of a patient with no catheter in the jugular vein developing Lemierre’s syndrome. Furthermore, while MSSA is a common isolate associated with central line-associated bacteremia, it rarely causes Lemierre’s syndrome. Using POCUS for DVT evaluation allows for rapid diagnosis of venous thromboembolism at bedside. We found two cases in the literature of Lemierre’s syndrome being diagnosed with POCUS.^4^,^5^

## CONCLUSION

Patients with central catheters, whether peripherally or centrally inserted, are at increased risk for both thromboembolism and septic thrombophlebitis. A high index of suspicion is required to make the proper diagnosis. Patients presenting to the ED with signs and symptoms of venous thromboembolism and infectious symptoms should undergo point-of-care ultrasound and sepsis evaluation to identify this rare but life-threatening pathology.
